# Opsonization-independent antigen-specific recognition by myeloid phagocytes expressing monoclonal antibodies

**DOI:** 10.1126/sciadv.adg1812

**Published:** 2023-09-01

**Authors:** Michael Neumaier, Sophie Giesler, Volker Ast, Mathis Roemer, Timo-Daniel Voß, Eileen Reinz, Victor Costina, Martin Schmelz, Elina Nürnberg, Stefanie Nittka, Aino-Maija Leppä, Ruediger Rudolf, Andreas Trumpp, Tina Fuchs

**Affiliations:** ^1^ Institute for Clinical Chemistry, University Medicine Mannheim, Mannheim, Germany.; ^2^ Mannheim Institute of Innate Immunoscience, Medical Faculty Mannheim of Heidelberg University, Mannheim, Germany.; ^3^ Department of Medicine I - Medical Center, Faculty of Medicine, University of Freiburg, Freiburg, Germany.; ^4^ Next Generation Sequencing Core Facility, Medical Faculty Mannheim of Heidelberg University, Mannheim, Germany.; ^5^ Institute of Nutritional Medicine, Department of Immunology, University of Hohenheim, Stuttgart, Germany.; ^6^ Department of Pain Research, Medical Faculty Mannheim, University of Heidelberg, Mannheim, Germany.; ^7^ Institute of Molecular and Cell Biology, Mannheim University of Applied Sciences, Mannheim, Germany.; ^8^ Division of Stem Cells and Cancer, German Cancer Research Center (DKFZ)-Center for Molecular Biology of Heidelberg University (ZMBH) Alliance, Heidelberg, Germany.; ^9^ Heidelberg Institute for Stem Cell Technology and Experimental Medicine (HI-STEM gGmbH), Heidelberg, Germany.

## Abstract

This report demonstrates a novel class of innate immune cells designated “variable immunoreceptor–expressing myeloids” (VIREMs). Using single-cell transcriptomics and genome-wide epigenetic profiling, we establish that VIREMs are myeloid cells unrelated to lymphocytes. We visualize the phenotype of B-VIREMs that are capable of genetically recombining and expressing antibody genes, the exclusive hallmark function of B lymphocytes. These cells, designated B-VIREMs, display monoclonal antibody cell surface signatures and regularly circulate in the blood of healthy individuals. Single-cell data reveal clonal expansion of circulating B-VIREMs as a dynamic response to disease stimuli. Live-cell imaging models suggest that B-VIREMs load their own Fc receptors with endogenous antibodies during vesicle transport to the cell surface. A first cloned B-VIREM–derived antibody (Vab1) specifically binds stomatin, a ubiquitous scaffold protein that is strictly expressed intracellularly, allowing Vab1-bearing macrophages to phagocytose cell debris without requiring prior opsonization. Our results suggest important antigen-specific tissue maintenance functionalities in these innate immune cells.

## Teaser

Immunorecognition revisited: smart phagocytes provide a sophisticated link of innate and adaptive functions in the immune system.

## INTRODUCTION

The immune system of vertebrates comprises two independent functional systems that are strictly separated with respect to their recognition mechanisms and their biological effector functions: an innate and an adaptive arm characterized by very distinct immunorecognition molecules for the detection of infectious agents (*1*, *2*), malignant cell growth (*3*, *4*), and tissue damage (*5*, *6*) or the surveillance of tissue homeostasis and immune tolerance (*7*, *8*). Specifically, the innate system comprises cells of myeloid differentiation that express a very limited set of genome-encoded, invariant pattern recognition receptors (PRRs) like the Toll-like receptors, CD14 or MD-2 (*9*). These receptors are readily available to recognize pathogen-associated molecular patterns or damage-associated molecular patterns as triggers for innate immune activation (*10*, *11*). In contrast, the phylogenetically younger adaptive immune system comprises lymphocytes that use somatic recombination of genome-encoded building blocks to generate, in a stochastic fashion, active genes coding for immunoglobulins (Igs) and T cell receptors (TCRs). Subsequently, the variable immunoreceptors are further improved in their antigen-binding efficiencies through antigen-driven hypermutations within the productive variable region genes. This enables the adaptive system to recognize and discern antigens with very high molecular resolution and affinity (*2*). In contrast to the innate immune system, this adaptive response is slow to develop, usually taking several weeks to respond effectively to a stimulus (*12*, *13*).

Over the past years, circumstantial evidence of partial mRNA coding for single-variable Ig genes has suggested that Ig expression by nonlymphocytic cells may occur in sample preparations of neoplastic cells (*14*), myeloid leukemia cell lines (*15*, *16*), and murine macrophages (*17*). Our group has reported the first full-length Ig heavy- and light-chain antibody sequences obtained from a single tumor-associated macrophage (*18*). However, beyond of the functional genomics level, the phenotype of the cells, which we designate “variable immunoreceptor–expressing myeloids” (VIREMs), has not been demonstrated. In addition, their progeny, the specificities of their variable immunoreceptors, and their biological functions remain entirely elusive so far.

Phagocytosis is a major effector function of the innate immune system allowing monocytes/macrophages, granulocytes, dendritic cells, and osteoclasts to engulf and digest particulate matter like bacteria, cell fragments, and microparticles (*19*, *20*). If target surfaces are decorated (opsonized) with antibodies, then they become “visible” to phagocytes through attachment and cross-linking of their cell surface Fc γ receptors (FcγRs) generating the specific trigger for opsonophagocytosis (*21*, *22*). In contrast, confronting phagocytes with increasing concentrations of nonimmune human serum are known to block their FcγR-mediated effector functions (*23*).

Here, we provide the first phenotypic evidence for B-VIREM cells expressing monoclonal antibodies, both morphologically and functionally, and characterize them at the molecular level. Genome-wide methylation studies and single-cell transcriptomics demonstrate the myeloid origin of B-VIREM with very close kinship to monocytes/macrophages while being developmentally distant from lymphocytes. B-VIREM cells expressing monoclonal antibodies can be visualized distinctively in the peripheral blood of healthy donors and patients, in part showing marked clonal expansions potentially related to health conditions. Furthermore, we identify the specific antigen of the first cloned B-VIREM–derived monoclonal antibody and provide a model for the functionality of endogenous antibody expression in myeloid cells simultaneously expressing Fc receptors (FcRs) to allow for an antigen-specific yet opsonization-independent way of innate phagocytosis. Our work establishes B-VIREM cells as a class of monocytes/macrophages expanding our fundamental understanding of distinct immune mechanisms in health and disease.

## RESULTS

### Visualization and characterization B-VIREM in PBMCs

**Fig. 1.**
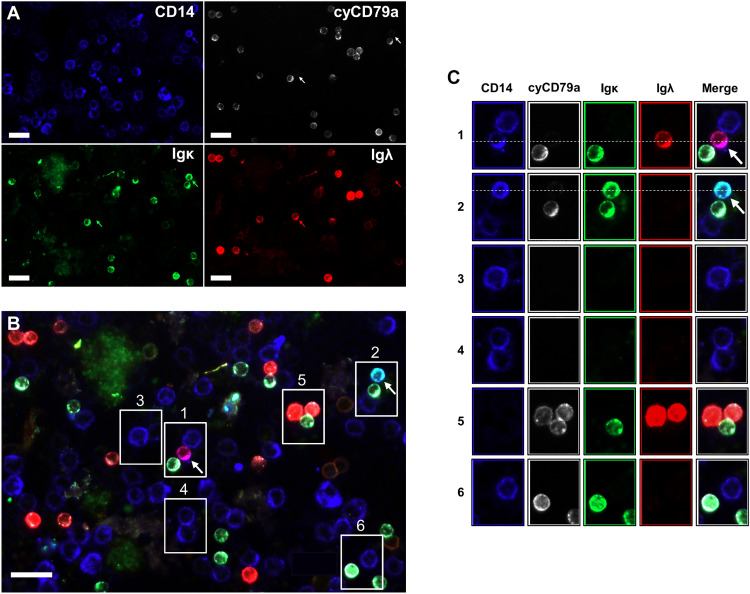
Phenotyping of antibody-expressing cells in the peripheral blood of healthy individuals identifies B-VIREM cells. (**A**) Immunofluorescence staining of peripheral blood mononuclear cells (PBMCs) using antibodies directed against CD14, cyCD79a, and Igκ and Igλ light chains presented in individual fluorescence channels. (**B**) Merged image of (A); boxed areas relate to images of interest and are presented in more detail; arrow positions as in (A). (**C**) Columns relate to the four fluorescent biomarkers used. Rightmost column taken to boxed areas in (B); rows 1 and 2 show antibody-expressing B-VIREMs highlighted by arrows and dashed lined across. Rows 3 and 4, CD14^+^ monocytic cells; row 5, cyCD79a^+^Igλ^+^ B cells and cyCD79a^+^Igκ^+^ B cell; row 6, CD14 ^+^ monocyte and cyCD79a^+^Igκ^+^ B cells. Scale bars, 20 μm.

Normal peripheral blood monocytes show a polyclonal Ig cell surface signature due to simultaneous binding of both kappa- and lambda-bearing antibodies through FcRs, resulting in mixed fluorescence (fig. S1A). Immunostaining of peripheral blood mononuclear cells (PBMCs) identified monocytes and B cells by CD14^+^ and cyCD79a positivity, respectively, and also determined the presence of antibodies by staining for kappa or lambda light chains (Fig. 1A). Figure 1B shows a superimposition of representative fluorescent images to distinguish regular CD14^+^ monocytes (blue) from B-VIREM. The latter show fluorescence staining in cyan (mix of blue and green) and magenta (mix of blue and red), which identifies these CD14^+^ monocytes as kappa- or lambda-expressing B-VIREM cells, respectively. Multicolor immunostaining consistently identified B-VIREM in PBMCs at frequencies of approximately 1.73 ± 0.48% among the CD14^+^ cells (fig. S1B and table S1). Figure 1C and fig. S1C show all B-VIREM cells identified in this study and allow for estimation of their frequencies and an assessment of the appropriate in-sample controls for sensitivity and specificity. Under conditions of high overexposure of Fig. 1B, B-VIREM cells stain positive for CD79A, an accessory protein for BCR signaling (fig. S1D). These results establish that B-VIREM cells are immunocytochemically distinct from B cells and regular CD14^+^ monocytes and are regularly found circulating in peripheral blood of healthy individuals.

**Fig. 2.**
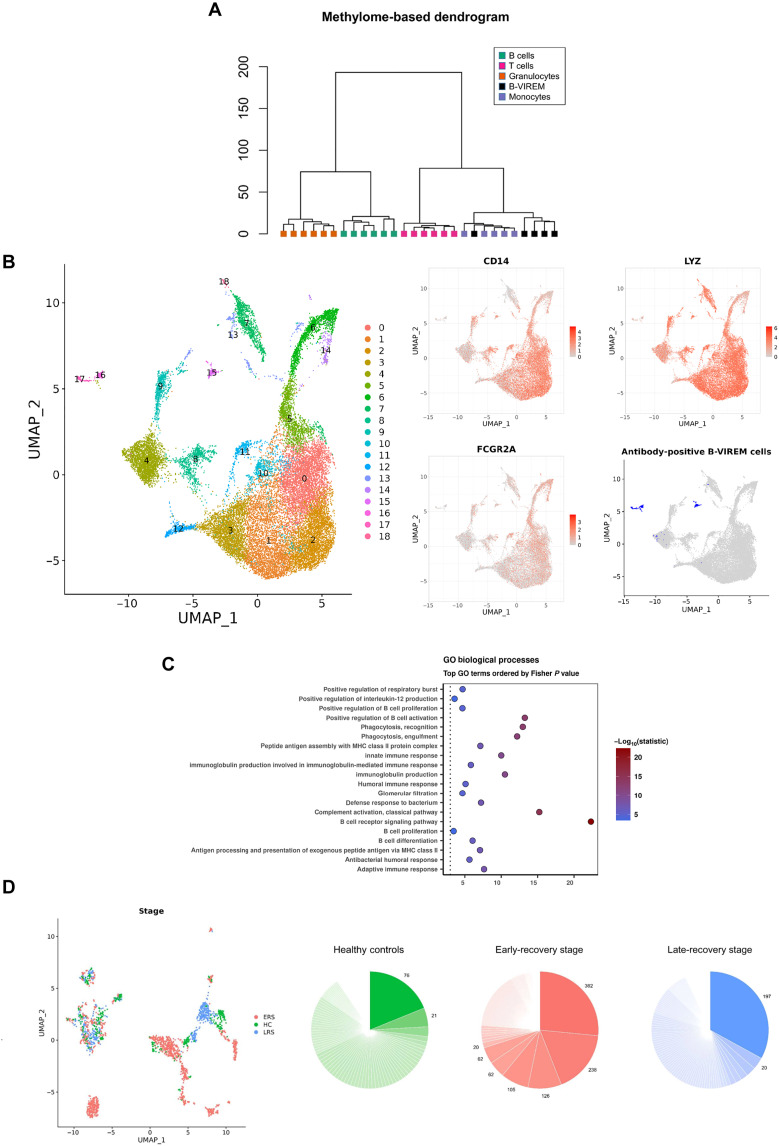
Single-cell mRNA analyses reveal B-VIREM cell populations in PBMCs. (**A**) Methylome analyses of fluorescence-activated cell sorting–sorted leukocytes from six healthy donors. A total of 10,652 DMRs from all possible pairwise comparisons between four cell populations (B cells, T cells, granulocytes, and monocytes) were used to cluster blood cell populations demonstrating that B-VIREMs are of closest kinship to monocytes. (**B**) Single-cell transcriptomic data of PBMCs (*n* = 4) were classified by V(D)J profiling. Monocytes/macrophages and B-VIREMs were integrated and subclustered using uniform manifold approximation and projection (UMAP) from clusters 0 to 3 (classical monocytes) and clusters 15, 16, and 17 (potential B-VIREM). Feature plots illustrate the expression of myeloid markers and Ig heavy- and light-chain expression. (**C**) Pathway enrichment analysis of B-VIREM cluster 15 compared to monocyte cluster 0. The top 20 differentially expressed Gene Ontology (GO) terms (*P* < 0.001) are shown. MHC, major histocompatibility complex. (**D**) Left: Single-cell analysis of B-VIREM in healthy controls (HCs) and in patients with coronavirus disease 2019 (COVID-19) from early-recovery stage (ERS) and late-recovery stage (LRS) in technical quadruplicates. Right: Ig heavy-chain clonotypes of antibody-expressing B-VIREM cells. The dark pie sections represent expanded clonotypes with numbers specifying factor of expansion. Total number of B-VIREM cells in each cell group: HC, 431; ERS, 1400; LRS, 667. For distribution of clonotypes, see table S2.

Leukocyte populations and B-VIREM cells were sorted from peripheral blood samples of healthy individuals and analyzed for their lineage affiliation using a genome-wide 850k methylome array defining differentiation-dependent intragenic and intergenic methylation patterns (Fig. 2A). This analysis reveals that B-VIREM cells differentiate within the myeloid lineage showing closest kinship to monocytes followed by granulocytes and are developmentally not related to lymphocytes.

Publicly available single-cell data of PBMCs from healthy donors allowed to cluster the RNA sequencing [single-cell RNA (scRNA)] profiles of all monocytic cells in the datasets. Subsequent V(D)J profiling of the scRNA profiles revealed three putative B-VIREM clusters (clusters 15, 16, and 17; Fig. 2B). Sample bias led us to exclude clusters 4, 16, and 17 from further analyses (fig. S2A) and further analyze cluster 15 accounting for approximately 0.8% of the cells. Gene Ontology analysis revealed that, together with classical innate pathways like phagocytosis and innate immune response, B-VIREMs showed marked enrichment of prominent adaptive immune pathways when compared to classical monocytes (clusters 0) (Fig. 2C). For example, the B cell receptor (BCR) signaling pathway was found to be most prominently activated, and alternatively spliced mRNA species for soluble and for membrane-bound Ig were identified in cluster 15, suggesting BCR expression. In line with this, B-VIREM and B cells express comparable ratios of membrane Ig, while plasma cells predominantly express soluble Ig (fig. S2B). In addition, different Ig isotypes appear to be expressed by B-VIREMs (fig. S2C). B-VIREMs show an approximately threefold higher rate of cells with unproductive Ig rearrangements compared to circulating B cells (fig. S2D). Together, the immunocytochemical phenotype, the transcriptional profile and pathway analyses, and the genome-wide methylome analyses unidirectionally point to the myeloid nature of B-VIREMs.

To address the question about potential dynamic changes of blood-borne B-VIREM in different health conditions, we analyzed the V(D)J single-cell datasets of a study of 14 PBMC samples (in technical quadruplicates) from patients with coronavirus disease 2019 (COVID-19) and from healthy individuals [healthy control (HC)]. The COVID-19 samples were taken in their early-recovery stage (ERS) and late-recovery stage (LRS), i.e., during the clinical virus-positive phase and the polymerase chain reaction (PCR)–negative post–COVID-19 phase, respectively (Fig. 2D) (*24*). From the HC, ERS, and LRS samples, we extracted full-length sequences of corresponding heavy and light chains of Igs coding for 116, 131, and 133 bona fide intact antibody clonotypes, respectively (table S2). The pie charts in Fig. 2D show that, of the total of 380 clonotypes, 29 showed different degrees of clonal expansions. These were most pronounced in the ERS stage with up to 362 cells in one sample expressing the identical antibody, while the HC group showed significantly fewer and lower-degree clonotype expansions (ERS versus HC, *P* = 0.033, Wilcoxon test). When clustering the B-VIREM clones, we observed two clusters not discriminatory for the HC, ERS, or LRS, while the expanded clusters coincided with the health conditions (Fig. 2D, left).

### Self-arming of FcR CD32A by endogenous antibody expression

**Fig. 3.**
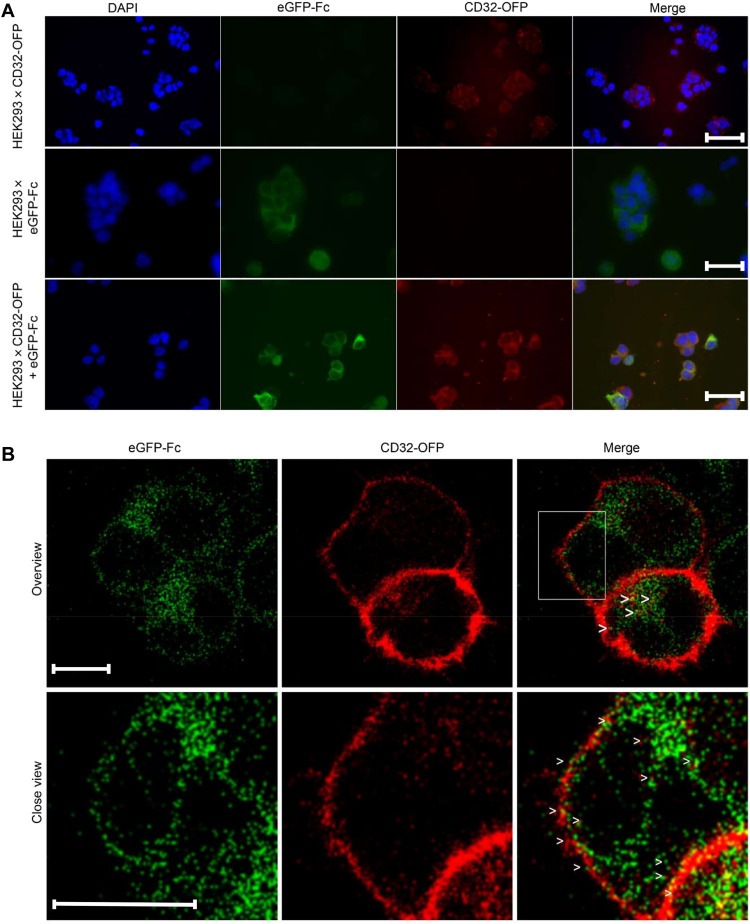
Simultaneously expressed Ig and FcR colocalize during intracellular vesicle transport and on the cell surface. (**A**) Fluorescence microscopy of human embryonic kidney–293 (HEK293) cells transfected with CD32–orange fluorescent protein (OFP) (top row), enhanced green fluorescent protein (eGFP)–Fc (middle row), and CD32-OFP + eGFP-Fc (bottom row) as indicated. Nuclear staining of ells previously grown on coverslips was done with 300 μM 4′,6-diamidino-2-phenylindole (DAPI). Single and merged fluorescence images as indicated. Scale bars, 50 μm. (**B**) Snapshot of the live-cell imaging video sequence of HEK293 cotransfected with CD32-OFP and eGFP-Fc. Double-fluorescent vesicles are highlighted by arrowheads in the cytoplasm, in the vicinity to the cell membrane and on the surface. Video sequences show intracellular transport of both proteins with 20% of mixed-fluorescence vesicles (movies S1 and S2; see also the Supplementary Materials). Scale bars, 5 μm.

We have coexpressed an FcγRIIA (CD32) fused to orange fluorescent protein (OFP; CD32-OFP) with an IgG-Fc domain fused to the C terminus of enhanced green fluorescent protein (eGFP) (eGFP-Fc) in human embryonic kidney–293 (HEK293) cells to investigate whether B-VIREM cells will self-arm their membrane FcRs with endogenously expressed Ig as suggested by the immunocytochemical images of the cells (Fig. 3A). Figure 3B shows cellular sections with vesicular transport of both molecules being simultaneously shuttled to the cell surface. Live imaging of several independent experiments with double transfectants demonstrated that around 20% of the vesicles in transit to the cell membrane were double positive (Fig. 3B, movies S1 and S2, and fig. S3D). Expression of CD32 and the functionality to bind to Ig-Fc domains were effective in HEK293 + CD32-OFP cells (fig. S3A). In double-transfected HEK293 cells (CD32-OFP^+^/eGFP-Fc^+^), the soluble eGFP-Fc fusion protein was not displaced from the surface FcR by polyclonal human IgG even at normal serum concentration (fig. S3B). In contrast, when CD32-OFP–transfected HEK293 cells were offered the eGFP-Fc fusion protein externally, its binding to the surface was efficiently competed for by increasing concentrations of polyclonal IgG as shown by diminishing green fluorescence staining (fig. S3B). These results demonstrate that coexpression of Ig and CD32 in cells simultaneously expressing both molecules allows efficient self-arming of the membrane FcR with monoclonal antibody and protects from displacement by external Ig. The results also indicate that, in the extravascular space, where IgG concentrations are approximately 13-fold lower, monoclonal antibody surface signatures will be stable on B-VIREMs (fig. S3C) (*23*).

### Identification of stomatin as the specific antigen of Vab1

**Fig. 4.**
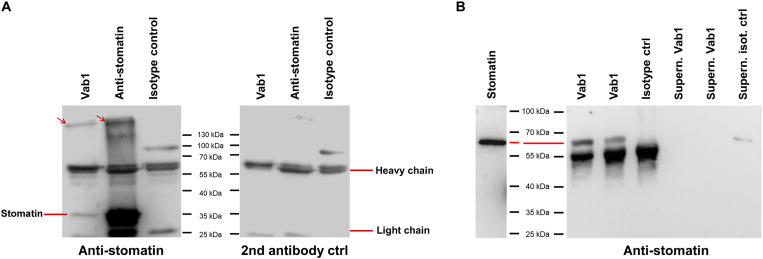
B-VIREM antibody identifies human stomatin as specific target by Western blotting. (**A**) Antigens were immunoprecipitated from erythrocyte membrane ghost preparations using Vab1, murine monoclonal anti-stomatin, and murine antibody isotype control as indicated. Left: Staining with murine anti-stomatin and horseradish peroxidase (HRP)–coupled rabbit anti-mouse antibody; the 35-kDa band corresponds to monomeric human stomatin; red arrows mark high–molecular weight moieties suggesting membrane complexes containing stomatin. Right: Staining with HRP-coupled rabbit anti-mouse antibody to detect unspecific binding; heavy and light chains detected by secondary antibodies are indicated. (**B**) Recombinant stomatin–glutathione *S*-transferase (GST) fusion protein (predicted molecular mass of 62 kDa) immunoprecipitated with Vab1 and human isotype control antibody followed by staining with murine anti-stomatin and HRP-coupled rabbit anti-mouse antibody. Left: Recombinant stomatin–GST (65 ng of stomatin). Right: Different amounts of stomatin were used for precipitations [lanes 1, 3, 4, and 6: stomatin (9 μg/ml); lanes 2 and 5: stomatin (4.5 μg/ml)]. Lanes 1 to 3, immunoprecipitates; lanes 4 to 6, supernatants of immune-depleted stomatin samples; lane 6, stomatin not precipitated from the solution by the human IgG1κ isotype control antibody.

As specific immunorecognition appears to be a key role in B-VIREMs, we have expressed the first myeloid-derived antibody reported (*18*) and studied its specificity to approach the cells’ biological functions. On the basis of a total of 24 immunoprecipitation experiments from human cell line extracts, PBMCs, and human erythrocytes followed by protein mass spectrometry (MS), we have identified stomatin, a strictly intracellular protein as the antigen specific for Vab1 in UniProt (table S3) (*25*, *26*). Western blot analysis of erythrocyte membrane ghost preparations immunoprecipitated by Vab1 and the commercially available monoclonal anti-stomatin antibody clone E5 demonstrate a protein band of 35 kDa corresponding to the molecular mass of monomeric stomatin and a high–molecular weight complex (Fig. 4A and fig. S4). Vab1 also identifies a recombinant stomatin–glutathione *S*-transferase fusion protein and depletes it from solution, while control IgG does not (Fig. 4B). As assessed by MS, 72 ± 5% of the proteins precipitated with both monoclonal antibodies were identical, and two-thirds are known to complex with stomatin in erythrocytes (table S4). Together, the data obtained by independent methods demonstrate that the monoclonal B-VIREM antibody Vab1 is specific for the intracellular scaffold protein stomatin.

### Mediation of phagocytosis by the monoclonal B-VIREM antibody Vab1

**Fig. 5.**
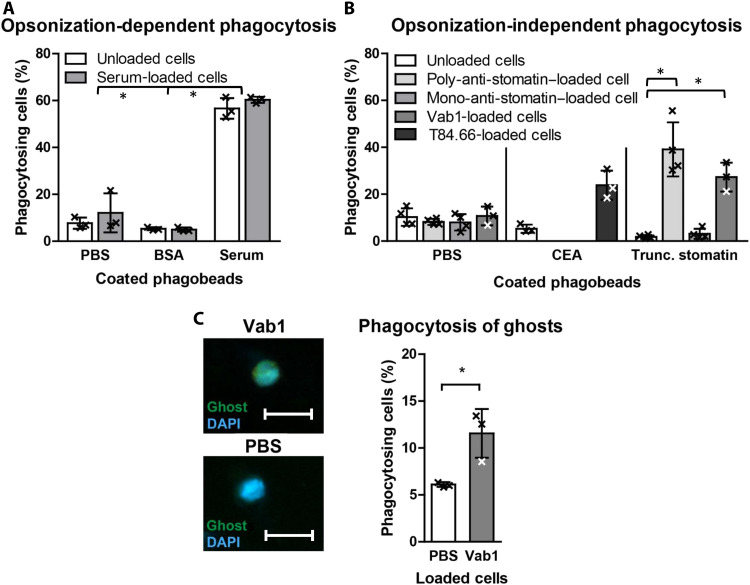
Phagocytosis of latex microbeads and erythrocyte membrane ghosts by plain and antibody-loaded myeloid cells. Each experiment used 10^5^ HL-60 phagocytes co-incubated overnight (o/n) at 37°C with latex beads (yellow-green fluorescence, 1-μm diameter) or with freshly prepared carboxyfluorescein diacetate succinimidyl ester–labeled erythrocyte ghosts. Results are presented as % phagocytosing cells and given as means with SD. (**A**) Coating of beads o/n at 4°C: PBS (uncoated), BSA (900 nM BSA), and serum (20 μg human serum). Plain or preloaded HL-60 phagocytes were used as indicated. *P < 0.01 (**B**) Coating of beads o/n at 4°C: PBS (uncoated), CEA (900 nM CEA), and truncated stomatin (900 nM recombinant stomatin lacking the 55 N-terminal amino acids). Preloading of HL-60 phagocytes with each antibody (2 μg/10^5^ cells) as indicated; *P < 0.05. (**C**) Phagocytosis of erythrocyte membrane ghosts by HL60 cells. Left: Images of HL-60 cells preloaded with Vab-1 (2 μg/10^5^ cells, top) or unloaded (PBS, bottom). Cells are counterstained with DAPI; magnification, ×40. Scale bars, 20 μm. Right: Results from three independent experiments; *P < 0.05.

We first confirmed that phagocyte cell line HL-60 predominantly expresses FcγRIIA (CD32) (fig. S5A). These cells can be successfully stripped and reloaded with polyclonal serum or monoclonal antibodies (fig. S5B). Opsonophagocytosis by HL-60 cells is efficient for serum-coated latex microbeads, while plain beads or bovine serum albumin (BSA)–loaded particles are not engulfed (Fig. 5A). HL-60 phagocytes preloaded with polyclonal anti-stomatin or with monoclonal Vab1 efficiently phagocytosed latex beads coated with a truncated recombinant stomatin lacking the N-terminal 55 amino acids (Fig. 5B). In contrast, the commercial monoclonal antibody clone E5 directed against the N-terminal stomatin domain does not provoke phagocytosis of the truncated stomatin demonstrating that the Vab1-specific epitope is not located in the N-terminal domain. HL-60 armed with the monoclonal anti–carcinoembryonic antigen (CEA) T84.1 antibody phagocytosed CEA-coated beads, while none of the other antibody-bearing cells did. Similarly, CD14^+^ cell preparations from five different healthy individuals previously loaded with anti-stomatin engulf stomatin-coated beads, while unloaded cells do not (fig. S5C). This confirms that the cells armed via their FcR do not require additional opsonization of antigen-positive surfaces. When using carboxyfluorescein diacetate succinimidyl ester–labeled erythrocyte membrane ghosts as a physiological phagocytosis substrate, Vab1-loaded HL-60 showed a 2.5-fold higher activity over unloaded cells (Fig. 5C). In contrast, phagocytosis of intact erythrocytes by anti-stomatin–loaded cells is not successful (fig. S5D).

## DISCUSSION

A central, long-standing paradigm in immunology posits that the strategies and mechanisms of immunorecognition used by the innate system and the adaptive system are fundamentally different and mutually exclusive, leading to a strict functional separation of the two arms. Specifically, the phylogenetically older innate immune system relies on genome-encoded preconfigured and invariant PRRs. In contrast, the younger adaptive system generates enormous repertoires of variable immunoreceptors in the form of antibodies and TCRs by stochastic recombination of independent genomic building blocks with subsequent antigen-driven clonal selections and refinement of their binding properties. Functionally, innate immune cells use a fast-responding recognition system with low antigen resolution, while the lymphocytes of the adaptive system provide antigen recognition at extremely high molecular resolution at the cost of a delayed immune response time for their generation and maturation. A very limited number of studies have reported partial sequences of isolated recombined Ig gene transcripts in nonlymphoid cells and cancer cell lines, mostly based on PCR amplification results (*15*, *16*, *18*, *27*). Using a self-developed single-cell technology, we have previously reported the first full-length light- and heavy-chain variable region genes coding for a bona fide intact monoclonal antibody obtained from a tumor-associated macrophage (*18*). However, phenotype and progeny, functionality, and biological significance of these cells have remained undetermined until now. Our report establishes a class of innate cells designated VIREMs that provide a link between the recognition functions of the innate and adaptive arms of the immune system.

The visualization of antibody-expressing VIREM (B-VIREM) by fluorescence microscopy now establishes these cells as a regular component in the blood of healthy individuals. The B-VIREM phenotype is easily distinguished from antibody-expressing B cells and from regular CD14^+^ monocytes by its additional monoclonal antibody cell surface signature exhibiting either kappa or lambda light-chain expression. We found approximately 1.7% B-VIREM cells among the CD14^+^ monocytes (Fig. 1B; fig. S1, B and C; and table S1). The investigation of leukocytes from healthy individuals using intra- and intergenic genome-wide methylome analysis that differentiates cell lineages of hematopoiesis (*28*) establishes B-VIREM cells as myeloid leukocytes with closest kinship to monocytic cells but unrelated to lymphocytes (Fig. 2A). The combined single-cell mRNA and V(D)J profiling data from four healthy donors show that B-VIREMs account for well-defined clusters among monocytes (Fig. 2B).

Gene Ontology pathway analyses show key B cell pathways being activated in B-VIREM in contrast to regular monocytes (Fig. 2C) consistent with the immunofluorescence and molecular data. For example, our data support the notion that B-VIREM will use B lineage markers associated with the antibody expression function. For example, our earlier data had shown that B-VIREM can express CD79A at the mRNA level (*18*), which is now confirmed. Specifically, fig. S1D shows that overexposure of the PBMC fluorescence staining in Fig. 1A demonstrates faint but discernable positivity in the antibody-expressing CD14^+^ monocytes, i.e., B-VIREMs. CD79A is an accessory molecule involved in signaling of the BCR until now associated with B cells. B-VIREMs express alternatively spliced Ig heavy-chain transcripts comprising the membrane exon required for the BCR. The proportion of mRNAs coding for the membrane and secreted antibody moieties is comparable to that of B cells. By comparison, plasma cells only show low expression of membrane-bound Ig heavy chain (fig. S2B), as expected.

One central question relates to the potential dynamics of B-VIREM in response to biological stimuli in health and disease. As a first approximation, we used uniform manifold approximation and projection (UMAP) clustering of independently published single-cell transcriptomic data obtained from peripheral blood samples of healthy individuals (HC) or patients in ERS and LRS of COVID-19. The samples show B-VIREM clusters that are indiscriminate for the three cohorts, while others display a patient- and stage-dependent differential clustering (Fig. 2D). Together, the B-VIREMs expressing full-length corresponding light- and heavy-chain genes of bona fide intact antibodies represent 380 different clonotypes (table S2), 29 of which were clonally expanded to different degrees as shown in the pie charts in Fig. 2D, e.g., with clonotypes of up to 362 cells expressing the same antibody. Both the number and the degree of the expanded clonotypes markedly differed in the three groups and were significantly pronounced in patients particularly during the ERS of the disease as compared the HCs. It appears highly likely that the observed expansions of B-VIREM clones are reactions to unknown biological and, in this COVID-19 study, disease-related stimuli encountered by these cells eventually that caused their dynamic skewing of B-VIREM repertoires seen in the blood. Considering that B-VIREMs are regular constituents in the blood of healthy individuals, this observation strongly suggests an important biological role of these cells in health and disease.

Ig expression in monocyte-derived cells raises questions about the mechanism enabling the monoclonal cell surface signature and the physiological function of B-VIREM. To investigate this, we resorted to a double-transfectant model of HEK293 cells allowing us to track OFP CD32 regularly expressed in monocytes and macrophages (*22*, *29*, *30*) without interference by a “cold” endogenous FcR. In contrast, the use of the THP-1 macrophage cell line was not feasible, because THP-1 would dilute the OFP-CD32 signal due to their endogenous FcR CD32, and they were also not stably transfected in our hands. Furthermore, we required an adherent cell system for easier live-cell imaging studies. Coexpression of the FcR CD32 and its Fc domain ligand in the double transfectants lead to coordinated transport of both molecules to the cell surface. Specifically, around 20% of vesicles display mixed fluorescence due to colocalization of the proteins (Fig. 3B and movies S1 and S2). These data explain the monoclonal surface signature of the B-VIREM phenotype as a result of CD32 occupied by their endogenous antibody Fc domain upon arrival at the surface. It is noteworthy that the monoclonal signatures are stable against the high concentrations of polyclonal serum IgG encountered in peripheral blood and, predictably, in the lower IgG concentration conditions within the extravascular space. This stability would be an important contributor for efficient opsonization-independent immunorecognition and the triggering of biological effector functions in this new class of innate immune cells. We hypothesize that the determination of specificities of B-VIREM antibodies is a key to approaching the functional roles of these cells. After recombinantly expressing the first functional B-VIREM–derived antibody (Vab1) recently identified in a tumor-associated macrophage (*18*), we found that Vab1 specifically recognizes stomatin, a strictly intracellular membrane-associated autoantigen that is ubiquitously expressed (Fig. 4). The specificity of Vab1 for stomatin was extensively confirmed. All commercial stomatin antibodies used in this study immunoprecipitated the antigen also as part of a high–molecular weight complex. This is consistent with its organization in a 9- to 12-mer oligomeric form as part of a multiprotein structure within lipid rafts (Fig. 4A and table S4) (*25*, *26*, *31*).

Phagocytes can identify targets in an antigen-specific fashion only, if these targets are opsonized by antibodies, which, in turn, require a paradigmatic preexisting B cell response (*19*). Antibody-decorated targets are subsequently bound by the cell’s membrane FcRs triggering opsonophagocytosis of the target. Our data strongly suggest a profoundly different mechanism for B-VIREM immunorecognition. Specifically, the endogenous concomitant expression of a monoclonal antibody and FcR leads to stable self-armament even during vesicle transport to the cell surface, thereby enabling the cell to exert specific binding to antigen-positive targets in an opsonization-independent fashion. In the case of Vab1, we demonstrate that the antibody-loaded cells will recognize particle matter, i.e., polystyrene and latex beads as well as membrane ghosts of destroyed erythrocytes known to be abundant with stomatin (*32*). For these experiments, we used HL-60 for its phagocytic capacity, although the cell line shows a more granulocyte-like differentiation (*33*, *34*).

**Fig. 6.**
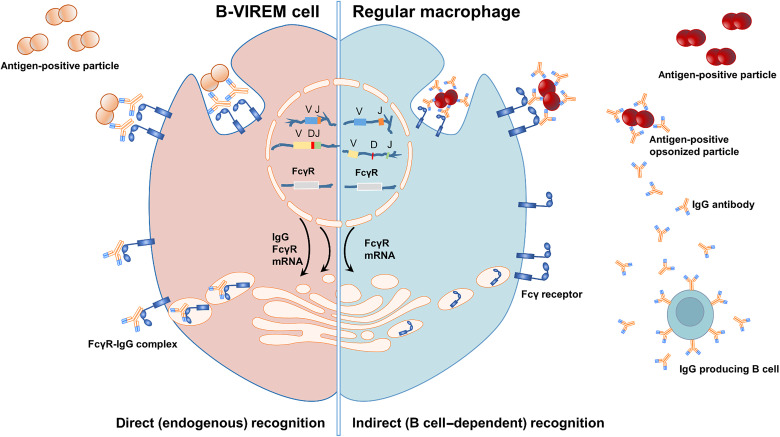
Schematic representation of antigen-specific immunorecognition and phagocytosis in myeloid cells. (**Left**) In a B-VIREM cell, the endogenous antibody and FcR are coexpressed and colocalize during transport to the cell surface leading to armed FcR. The specific antigen is directly recognized without requiring prior opsonization leading to direct phagocytosis. (**Right**) Only FcRs are expressed, and receptors transit to the cell membrane. To recognize immunogenic particles, opsonization with B cell–dependent Ig is required to cluster FcRs and leads to activation of phagocytosis of the targeted particles.

Specific immunorecognition of the strictly intracellular stomatin by a specific B-VIREM may allow antigen-specific “injury sensing” to trigger the clearance of damaged cells (*7*, *8*) without the need of prior B cell immunity. We speculate that such a B-VIREM function would aid in low-threshold restoration of tissue homeostasis and repair. We consider B-VIREM a prveiously unknown class of “smart phagocytes” capable of linking antigen-specific immunorecognition with innate biological effector functions parallel to, and even independent of, the lymphoid system (Fig. 6). Future studies need to investigate the spectrum of B-VIREM immunorecognition in detail. For example, this function may be part of an armory of tools required by complex organisms to maintain their bodily integrity at a low immunological activity threshold and contribute to the phagocytic code very recently reviewed by Cockram and colleagues (*35*). It is intriguing that, very recently, a concept called “normality sensing” has been proposed for tissue surveillance as a cellular immune function of γδT cells (*36*). Injury sensing could be a complementary innate function. Closely related to monocytes/macrophages, these cells designated B-VIREM are predicted to be capable of the antigen-specific immunorecognition of the B cell system combined with the instantaneous effector capabilities of innate effector functions.

## MATERIALS AND METHODS

### Patient samples

Tissue samples were obtained from the University Medicine Mannheim Biobank archive with written informed consent from the patients. Human buffy coats were provided by the German Red Cross Blood Donor Service Baden-Wuerttemberg Hessen. All blood specimens were anonymized. The study was approved by the local Ethics Committee II at the Medical Faculty Mannheim of Heidelberg University (2017-548 N-MA and 2014-562 N-MA).

### Cell culture and PBMC isolation

Human HL-60 (RRID:CVCL_0002) were cultured in RPMI (Merck, Burlington, USA) supplemented with 10% fetal bovine serum (FBS) (FBS.S0615; Bio&SELL) or X-VIVO-15 (Lonza). HEK293 cells (RRID:CVCL 0045) were cultured in Dulbecco’s modified Eagle’s medium (Thermo Fisher Scientific) supplemented with 10% FBS. Cells were cultured at 37°C, 5% pCO_2_, and >95% relative humidity and passaged two to three times per week to a maximum of 25 times. No used cell line is listed as “commonly misidentified cell line” by the International Cell Line Authentication Committee. In addition, cell lines were authenticated using the Multiplexion Multiplex Cell Authentication and Contamination tests (*37*, *38*). PBMC isolation was performed by gradient centrifugation (Ficoll-Paque PLUS) of human buffy coats. Erythrocytes were lysed by hypotonic shock, and the cell preparations were routinely counted in a Sysmex XP-300 hematocytometer (Sysmex).

### Immunofluorescence

PBMCs were isolated from heparinized peripheral blood samples of healthy volunteers, and ~5 × 10^5^ PBMCs were used for each staining. The cells were prepared for cytoplasmic staining with FIX&PERM (Nordic-MUbio) according to the manufacturer’s protocol. For staining of the permeabilized cells, the following antibodies were used: CD14-BV421 (clone M5E2; BD Biosciences), the multiMix dual-color reagent (kappa light chain–fluorescein isothiocyanate + lambda-phycoerythrin; Agilent-Dako), and cyCD79a-allophycocyanin (clone HM57; Agilent-Dako). The stained cells were mixed with heat-inactivated human serum at a 1:1 ratio, and cytospins (40*g* for 10 min) were prepared and analyzed on the Zeiss Axio Observer Z1.

### Single-cell sequencing and analysis

We obtained single-cell gene expression and V(D)J immune profiling datasets for two HC samples (denoted HC1 and HC2) with four technical replicates each (*24*). In addition, two PBMC data samples from healthy human donors (denoted 10X_10k and 10X_20k were downloaded from 10X Genomics (https://support.10xgenomics.com). To address technical artifacts of microfluidics-based systems and to reduce the risk of potential multiplet cell bias, we used four different detection tools: Scrublet (*39*), DoubletDetection (*40*), DoubletFinder (*41*), and SCDS (*42*). We excluded all cells, which were concordantly predicted as doublets by at least three prediction tools.

We used the Seurat R package (version 4.0.3) to analyze single-cell gene expression datasets (*43*). For quality control, we eliminated genes, which were detected in less than three cells and cells with less than 200 genes with nonzero counts. We further excluded all cells having more than 20% of mitochondrial gene counts.

Ig-positive cells were determined by integrating the V(D)J immune profiling datasets. In detail, we filtered for high-confidence, full-length, and productive Ig contigs and identified unique cells as V(D)J-positive cells if they expressed at least one Ig heavy chain (*IGH*) and at least one IG light chain (*IGkappa* or *IGlambda*). The four datasets were integrated and subsequently clustered using the UMAP method. Monocyte and macrophage clusters were identified using the expression profile of the markers *CD14*, *LYZ*, *S100A8*, and *S100A9*. A subset of all V(D)J-positive cells was extracted and reclustered in a separate analysis. Here, potential B-VIREM cells were identified using the expression level of the four monocytic markers and the formation of a distinct cluster. Last, monocytes and macrophages as well as B-VIREM were integrated and reclustered. Pathway enrichment analysis was performed using the TopGO R package (*44*), and cell type prediction was done with celldex (*45*).

### Clonotype expansion analysis

For the B-VIREM clonotype analysis, we accessed the complete single-cell gene expression and V(D)J immune profiling dataset from Wen *et al.* (*24*). For every patient group (ERS, LRS, and HC), we integrated the respective patient datasets (in quadruplicates) and identified B-VIREM as described above. Within these B-VIREMs, clonotypes for both heavy and light chains were identified by their full-length amino acid sequence according to their V(D)J data.

### Analysis of IGHM splice variants

To distinguish the secreted and membrane-bound *IGHM* transcript variants in B-VIREM cells, we investigated the expression of specific *IGHM* splice forms on the single-cell level. The DNA sequences of the *IGHM* exon 4, the *CHS* region, and the *M1* region were downloaded from imgt.org (*46*). The sequences of exon 4 and the *CHS* region representing the secreted form as well as exon 4 and *M1* region expressing the membrane-bound variant were joined. In addition, the *M1* region and the adjacent intron were combined to represent the pre-mRNA. Cellranger mkref was used to build a custom reference package. FASTQ raw reads were mapped on the custom reference by using the CellRanger count command. In the resulting bam file, reads overlapping the interface of exon 4 and *CHS* region and of exon 4 and *M1* region at position 330 were counted with the SAMtools view command. For the intron–M1 region, all reads covering position 490 were considered. Barcodes to identify specific cells were extracted from the CR tag of the reads header and summarized per sample.

### Analysis of productive and unproductive V(D)J antibody chains

We evaluated the BCR-V(D)J data from Wen *et al.* (*24*) and extracted all antibody chains that were positive for all of the binary attributes “is_cell,” “full_length,” “productive,” and “high_confidence.” Antibody chains that missed at least one criterion were considered as unproductive. Then, all cells having at least one productive heavy chain (*IGH*) and one productive light chain (*IGL* or *IGK*) were filtered. All cells missing this condition were regarded as cells expressing nonproductive antibodies.

### Methylome analyses

Lymphocytes, monocytes, granulocytes, and B-VIREM populations were fluorescence-activated cell sorting–sorted from peripheral blood samples of six healthy donors using the BD FACSMelody cell sorter (BD Biosciences). Lymphocytes and monocyte populations were sorted from PBMCs isolated by density gradient centrifugation (Ficoll-Paque PLUS), and granulocytes were sorted from the pellet sediment after Ficoll gradient centrifugation. Forward scatter (FSC) and sideward scatter (SSC) singlets were gated, and consecutive gating steps were applied to generate highly pure cell populations. The following sorting strategy and markers were used: B cells, CD14^−^CD19^+^; T cells, CD14^−^CD2^+^; monocytes, CD19^−^CD14^+^; B-VIREMs, CD19^−^ CD14^+^Igκ^+^Igλ^−^ or CD19^−^CD14^+^Igλ^+^Igκ^−^; and granulocytes, CD14^−^CD15^+^. After sorting, DNA was isolated using the QIAmp DNA Micro Kit (Qiagen) following the manufacturer’s protocol. The samples were then subjected to total methylome analysis using the Infinium Methylation EPIC BeadChip 850K array (Illumina). The work was carried out by the DKFZ Genomics and Proteomics Core Facility of the German Cancer Research Center, Heidelberg.

### Illumina infinium human methylation EPIC 850k bead array analysis

Raw intensity files were obtained using minfi package [Minfi 1.34.0; (*47*)] to calculate methylation ratios and the log_2_ methylation ratios (Beta values and *M* values, respectively). The data were normalized using Illumina preprocessing method implemented in minfi. Several quality control measures were applied to check for arrays with low quality. Median methylated and unmethylated signals were calculated for each array; detection *P* < 0.05 and badSampleCutoff = 10.5 were used to remove low-quality arrays. Probes that failed in one or more samples or contained an annotated single-nucleotide polymorphism at the single-base extension or CpG sites were removed (95,170 probes removed). Minfi 1.34.0 was used. Bump hunting method was applied to identify differentially methylated regions (DMRs) in EPIC 850k array [Bumphunter 1.30.0; (*48*)]. A maximum of 30,000 candidate bumps were used to determine the cutoff when finding DMRs (2.1 used with *M* values as input). Statistical significance was assigned by bootstrapping, and the familywise error rate (FWER)–adjusted *P* value cutoff used for downstream analysis was 0.05. To assign closest kinship of B-VIREM cells to normal blood cell populations, mean methylation value of each normal blood cell epigenetic signature (10,652 DMRs) for B-VIREM/normal blood cells (methylation profile) was retrieved. hclust function with ward method in R was used to generate the cluster dendrogram analysis.

### Cloning

The eGFP–open reading frame was PCR-amplified with primers including endonuclease cleavage sites for Eco RV (5′-agcttgcgcgatatcagtgagcaagggcgaggag-3′) and Nco I (5′-tattgcaccatggacttgtacagctcgtccatgc-3′) and removing start and stop codons using the *pEGFP-N1* (Clontech, Takara Bio) plasmid as template. For subcloning, the *pFUSE-hIgG1-Fc2* (*pfuse-hg1fc2*; InvivoGen) plasmid and the purified amplicon were both digested with Eco RV and Nco I for 2 hours at 37°C, following heat inactivation for 20 min at 80°C. Ligation was carried out by the Quick Ligation Kit (vector, 50 ng; insert, 75 ng; volume, 20 μl) as suggested by the manufacturer [New England Biolabs (NEB)]. The ligation mix was used to transform OneShot TOP10 cells (Thermo Fisher Scientific), and bacteria were plated on agar plates containing zeocin (50 μg/ml) and incubated overnight at 37°C. Several clones were picked, expanded, and isolated with the QIAprep Spin Miniprep Kit (Qiagen). Recombinant clones were identified by double restriction with Eco RI and Bgl II, and the integrity of their inserts was verified by DNA sequencing (Eurofins Genomics).

### Transfection and selection

For transfection of HEK293 cells, endotoxin-free plasmid DNA was linearized using Ase I (*pEGFP-N1*), Not I (*eGFP-Fc*), or Mfe I (pCD32-OFP (*pCMV3-CD32a-OFPSpark*, SinoBiological) and purified. HEK293 cells were transfected using the SF Cell Line 4D-NucleofectorTM X Kit L and the 4D-NucleofectorTM X Unit (Lonza) according to the manufacturer’s cell line protocol. Twenty-four hours after transfection, the medium was exchanged to medium supplemented with geneticin (1 mg/ml; *pEGFP-N1*), zeocin (100 μg/ml; *eGFP-Fc*), or hygromycin B (50 μg/ml; *pCD32-OFP*). Transfected HEK293 cells were analyzed by microscopy (TCS SP8 DLS Leica inverted Microscope DMi9 CS Bino DLS; Leica Microsystems). Live-cell imaging of transfected HEK293 cells was performed using a Leica TCS SP8 confocal microscope equipped with HCPL APO 20×/0.75 IMM CORR objective with 488- and 561-nm lasers.

### Preparation of erythrocyte ghosts and cell/tissue lysates

We prepared erythrocyte membrane ghosts as reported previously (*49*). In short, after Ficoll gradient centrifugation, erythrocytes were resuspended in 50 ml of washing buffer (isotonic NaCl 0.9% in 10 mM tris-HCl and 0.1 mM EGTA) and centrifuged at 1000*g* for 10 min at 15°C. After washing twice, erythrocytes were lysed in 37 ml of ice-cold sodium Hepes buffer [2.5 mM Hepes (pH 7.4) adjusted with NaOH and 0.1 mM EGTA] and centrifuged at 40,000*g* for 70 min at 4°C. The preparations were washed twice in 50 ml of phosphate-buffered saline (PBS) and centrifuged at 1000*g* for 10 min at 15°C, and the erythrocyte ghosts were resuspended in 1 ml of PBS and filtered through a 40-μm filter.

### Generation of recombinant antibodies

The B-VIREM antibody Vab1 was recombinantly expressed on the basis of cloned variable heavy chain (VH) and variable light chain (VL) sequences (*18*) in HEK293 cells as a IgG1/κ full-length Ig and provided as lyophilized powder (Syd Labs Inc.).

### Immunoprecipitation

Total protein lysates [*n* = 24: PBMCs (*n* = 10), erythrocytes (*n* = 6), HepG2 (*n* = 2), colon tumor tissue (*n* = 2), normal colon tissue (*n* = 2), and esophagus tumor tissue and normal esophagus tissue (both *n* = 1)] were prepared in radioimmunoprecipitation assay buffer. To desalt, buffer was exchanged by 30 min of centrifugation through Vivaspin 6 column (molecular weight cutoff, 5 kDa; GE Healthcare). To reduce nonspecific protein interactions, the lysates were subsequently precleared on Pierce Spin columns (Thermo Fisher Scientific) with an agarose matrix for 30 min at 4°C. Immunoprecipitations of stomatin were performed using 10 to 20 μg of Vab1 or the murine monoclonal anti-stomatin antibody (clone E5, Santa Cruz Biotechnology). Recombinant human IgG1κ clone (Bio-Rad) and purified murine IgG2bκ isotype control (BD Pharmingen) were used as control antibodies. Antibodies were diluted to concentrations of 50 μg/ml and incubated with protein A–coated magnetic Dynabeads (Thermo Fisher Scientific) for 30 min at room temperature and were subsequently washed with PBS. The desalted and precleared lysates were added to the beads at concentrations of 0.33 to 1.25 mg of protein per sample and incubated overnight at 4°C under constant movement. Beads were recovered and were washed three times in PBS/0.02% Tween 20 (v/v). The precipitated proteins were eluted in Laemmli buffer.

### Western blotting

Primary antibodies (rabbit anti-GFP) and murine monoclonal anti-human stomatin were used at indicated concentrations overnight at 4°C; secondary horseradish peroxidase–conjugated antibodies were incubated at indicated concentrations for 2 hours at room temperature. Immunoblots were developed using the Amersham ECL Prime Western Blotting Detection Reagent (GE Healthcare) by the ChemiDoc XRS+ Imager (Bio-Rad).

### Protein preparation and MS

Bands of interest were excised from the SDS–polyacrylamide gel electrophoresis separations and prepared for MS analyses. In short, the dried peptide samples were redissolved in 0.1% trifluoroacetic acid and loaded onto a C18 precolumn (Acclaim; Thermo Fisher Scientific) using an RSLCnano HPLC system (Thermo Fisher Scientific), from which they were separated with an aqueous-organic gradient using a flow rate of 300 nl/min. We used electrospray injection into an LTQ Orbitrap XL mass spectrometer (Thermo Fisher Scientific) using a Triversa Automate (Advion) as ion source. Each scan cycle consisted of one Fourier transform mass spectrometry (FTMS) full scan and up to seven ion trap mass spectrometry (ITMS)–dependent tandem MS (MS/MS) scans of the seven most intense ions. Dynamic exclusion (30 s), mass width [10 parts per million (ppm)], and monoisotopic precursor selection were enabled. All analyses were performed in positive ion mode. Extracted MS/MS spectra were searched against the human Uniprot database using the PEAKS search engine (Bioinformatics Solutions Inc.) accepting common variable modifications, one missed cleavage site, a peptide tolerance of ±10 ppm, and MS/MS tolerance of ±0.5 Da. All protein identification experiments were carried out using the corresponding decoy database and a false discovery rate of 1%. Further details as described elsewhere (*50*).

### Phagocytosis assay

Green-yellow fluorescent latex beads (10^7^ beads) with (1 μm; Merck) were coated with 900 nM BSA (NEB), 20 μg of human serum obtained from healthy individuals, 20 μg of mouse serum (Merck), 900 nM recombinant stomatin (Abcam or ProSpec), or 900 nM CEA (provided by J. E. Shively, City of Hope National Cancer Center) overnight at 4°C. Subsequently, coated beads were centrifuged, washed twice in PBS, resuspended in PBS (final concentration: 2.5 × 10^5^ beads/μl), and stored in the dark for <7 days at 4°C. HL-60 cells (10^6^; undifferentiated), HL-60 dimethyl sulfoxide (DMSO) (3 day differentiation with 1.3% DMSO), or primary CD14^+^–magnetic-activated cell sorting (Miltenyi Biotec) isolated monocytes were coated with 20 μg of polyclonal rabbit anti-human stomatin (Biomol), murine monoclonal anti-human stomatin, and Vab1 or anti-CEA antibodies (T84.1- and T84.66-containing cell culture supernatants), respectively, for 1 hour at 4°C in PBS. Cells were washed twice with PBS. Phagocytosis assay was performed in black 96-well clear bottom plates (Greiner Bio-One) with serum-free medium, beads (7 × 10^6^) or erythrocyte ghosts (7 × 10^6^), and cells (5 × 10^4^) added to the plate in final volumes of 180 μl per well followed by incubation for 3 hours or overnight at 37°C, 5% pCO_2_. Sytox blue (Thermo Fisher Scientific) was added, and phagocytosis was measured by flow cytometry using a FACSCanto II (BD Biosciences).

### Statistical analysis

Results represent means ± SD, unless otherwise indicated. Statistical significance between groups was determined by unpaired Student’s *t* test or analysis of variance (ANOVA) and Bonferroni or Dunnett’s correction for multiple comparisons.
