# Supplementary material for: Opsonization-independent antigen-specific recognition by myeloid phagocytes expressing monoclonal antibodies
Source: Sci Adv. 2023 Sep 1;9(35):eadg1812. doi: 10.1126/sciadv.adg1812 (PMC11314243; doi:10.1126/sciadv.adg1812)
Supplement: Supplementary file 1 — Figs. S1 to S5 Tables S1, S3, and S4 Legend for table S2 Legends for movies S1 and S2 [file sciadv.adg1812_sm.pdf]

Science Advances
AAAS

Supplementary Materials for

Opsonization-independent antigen-specific recognition by myeloid phagocytes expressing monoclonal antibodies

Michael Neumaier *et al.*

Corresponding author: Michael Neumaier, michael.neumaier@medma.uni-heidelberg.de

Sci. Adv. **9**, eadg1812 (2023)
DOI: 10.1126/sciadv.adg1812

The PDF file includes:

Figs. S1 to S5
Tables S1, S3, and S4
Legend for table S2
Legends for movies S1 and S2

Other Supplementary Material for this manuscript includes the following:

Table S2
Movies S1 and S2

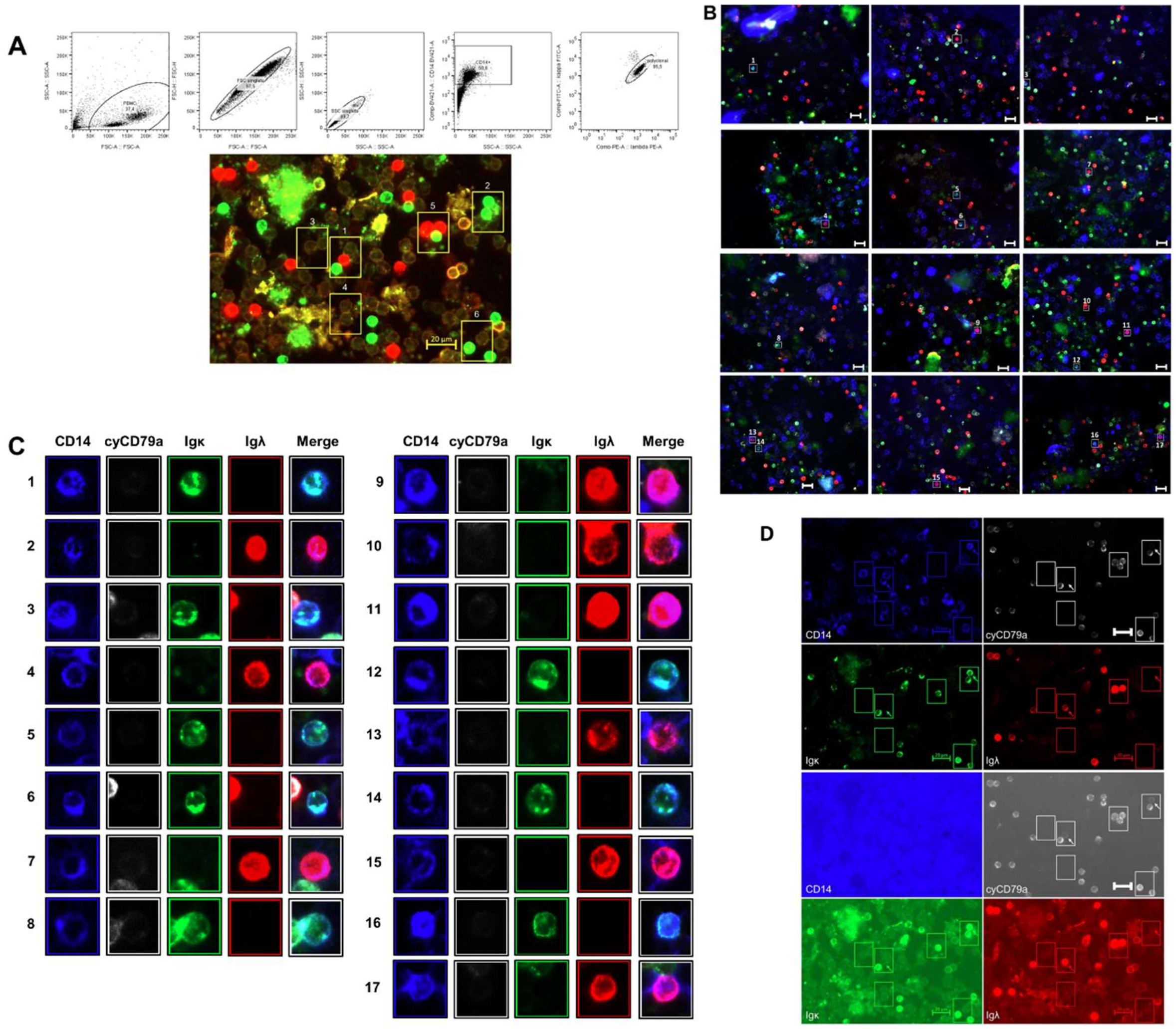

fig. S1. Immunochemical phenotyping of antibody-expressing cells in the peripheral blood of healthy individuals identifies B-VIREM cells. (A) Staining with Igκ (green) and Igλ (red) antibodies **(top)** Gating strategy of PBMC from a healthy donor to identify immunoglobulin surface staining in CD14+ monocytes. Cells showing polyclonal Igκ+ and Igλ+ staining are highlighted by a black circle. **(bottom)** Fluorescence image from Fig. 1B of the Igκ and Igλ channels digitally amplified and merged to allow better visibility of the signal. Boxes indicate the same cells as in Fig. 1B. **(B)** Merged images of additional micrographs from immunofluorescence stainings of PBMC using antibodies directed against CD14, cyCD79a, Igκ and Igλ; Areas of interest are boxed and presented in detail in Fig. S1C. Scale bar= 20µm. **(C)** As marked in Fig. S1B, B-VIREM cells are displayed in each single fluorescence channel as well as the merged image. **(D)** Immunofluorescence staining of PBMC as shown in Fig. 1A; top images under optimal exposure conditions, bottom images under overexposed conditions to show possible cyCD79a expression; fluorescence channels for CD14, Igκ and Igλ are shown as comparison for the extent of overexposure. Scale bar= 20 µm.

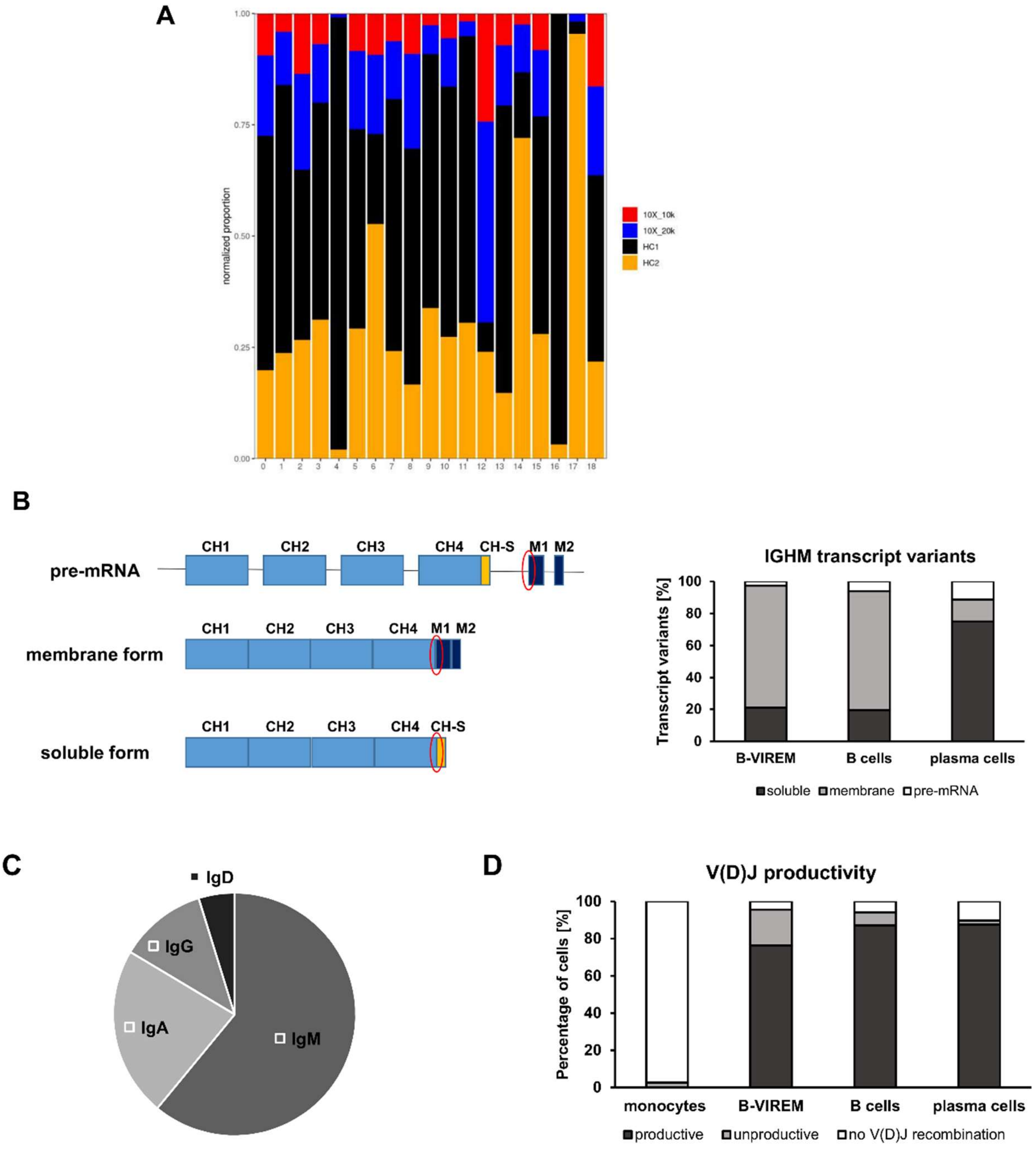

fig. S2. Single cell transcriptomic analysis in PBMC of healthy donors. (A) PBMC of four healthy donors were analysed by single cell RNASeq and V(D)J profiling. The graph shows the sample distribution of all clusters. Due to sample bias, clusters 4, 16 and 17 were excluded from further analysis. **(B) Left**: Scheme of alternatively spliced IGHM transcripts and pre-mRNA species identified on the basis of exon/exon and intron/exon junctions as shown by the red circles. **Right:** The distribution of the three transcript variants of the IGHM constant regions were analysed in B-VIREM, B cells and plasma cells. **(C)** Distribution of immunoglobulin isotypes of B-VIREM cells in cluster 15. **(D)** Single cell data of monocytes, B-VIREM, B cells and plasma cells were analysed for productive and unproductive V(D)J recombinations.

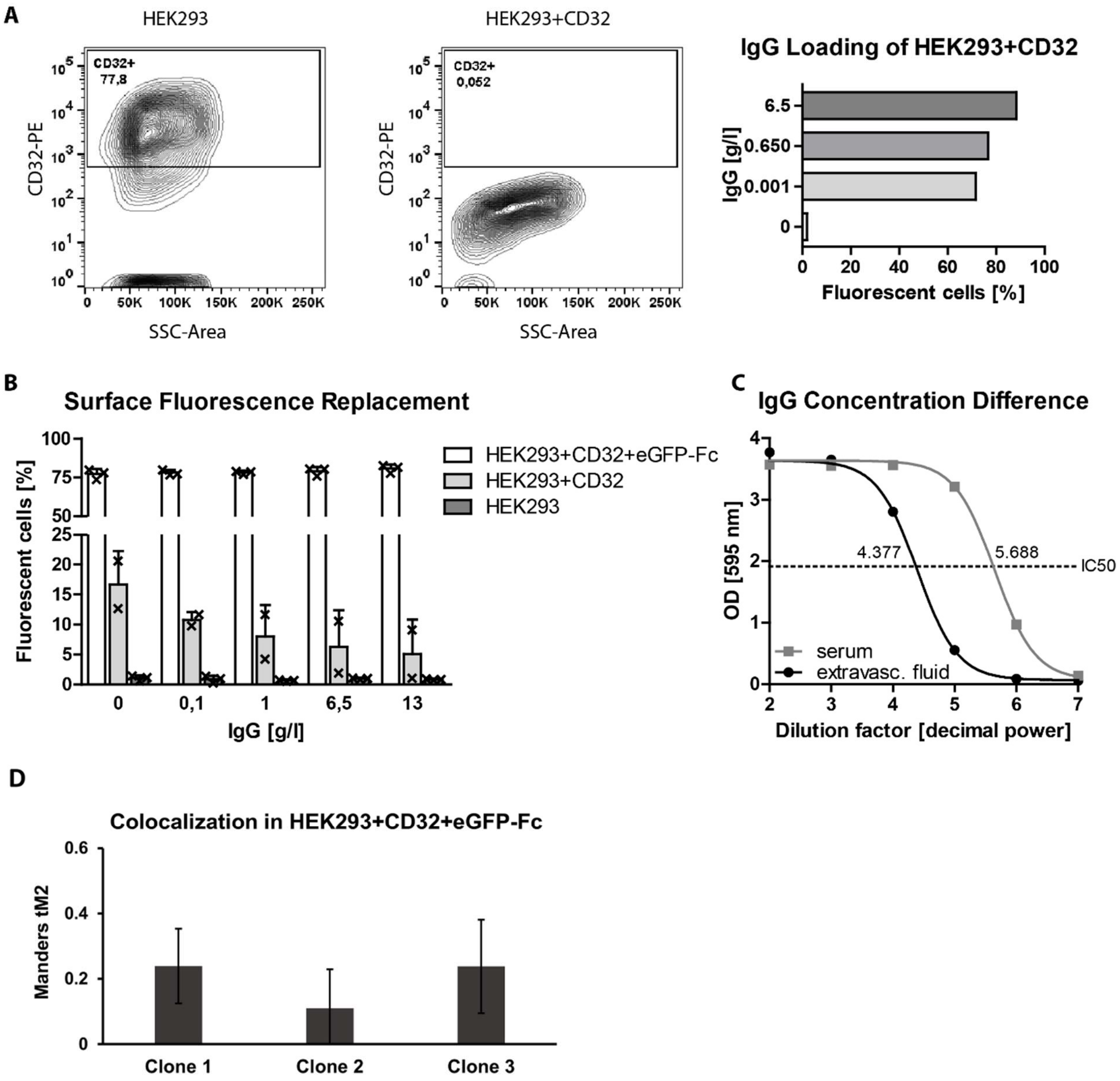

fig. S3. Transfection models to study FcγR binding to immunoglobulin Fc-domain and stability of complex. (A) Left panel: 78% of CD32-OFP transfected HEK293 display CD32 as detected by CD32-PE antibody. **Middle panel:** Normal HEK293 are not stained by CD32-PE antibody. **Right panel:** CD32-transfected HEK293 cells show binding of human serum immunoglobulin at various concentrations as shown by staining with Alexa488-labeled IgG-Fab2 fragment. **(B)** a) Stability of the CD32/eGFP-Fc complex on the cell surface of double transfected HEK293+CD32+eGFP-Fc cells in the presence of increasing concentrations of human serum IgG. b) Binding of eGFP-Fc offered externally to HEK293+CD32 cells is inhibited by serum IgG in a concentration-dependent manner. c) Untransfected HEK293 cells do not bind eGFP-Fc. Results from three independent experiments are presented as Mean±SD. **(C)** Estimation of relative concentration differences of serum IgG and extravascular fluid by solid-phase ELISA. Extravascular fluid harvested from microdialysis tubes implanted subcutaneously was coated on a 96-well plate in ten-fold dilutions and detected by HRP-conjugated goat anti-human IgG antibody. The microdialysate showed a 13-fold lower IgG concentration than the donor´s serum IgG (14.6g/l) as determined by EC50 calculation using Prism 5 (GraphPad Inc.) software**. (D)** Colocalization analysis of three independent clones from

the double transfectant cell line HEK293+CD32+eGFP-Fc. The Manders coefficient tM2 was calculated of the peri-membranous region from 10 time-lapse videos of each clone. For colocalization analyses the Coloc2 plugin from ImageJ was used. Results are shown as mean±SD.

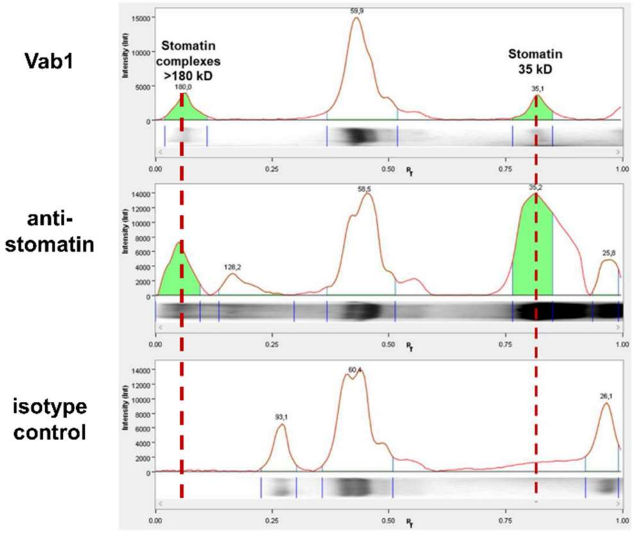

Lane	Mol wt (kDa)	Band Label	Relative Front (Rf)
Vab1	>180	Stomatin complexes	0.07
	59.9	Ig heavy chain	0.44
	35.1	Stomatin	0.82
anti-stomatin	>180	Stomatin complexes	0.06
	128.2	Unknown	0.17
	58.5	Ig heavy chain	0.45
	35.2	Stomatin	0.82
	25.8	Ig light chain	0.97
isotype control	93.1	Unknown	0.28
	60.4	Ig heavy chain	0.43
	26.1	Ig light chain	0.97

fig. S4. Densitometric analyses of immunoblots

Molecular weight analysis of the Western blot shown in Figure 4A. Immunoprecipitated erythrocyte lysates were separated by SDS-Page and detected in the Western blot with the indicated antibodies. Analysis demonstrates the molecular weight of monomeric stomatin to be 35.1kD +/- 0.1kDa (lanes 1, 2) and the molecular weight of stomatin complexes to be >180 kDa. In lane 3, no bands of this size can be detected in agreement with a specific depletion of stomatin.

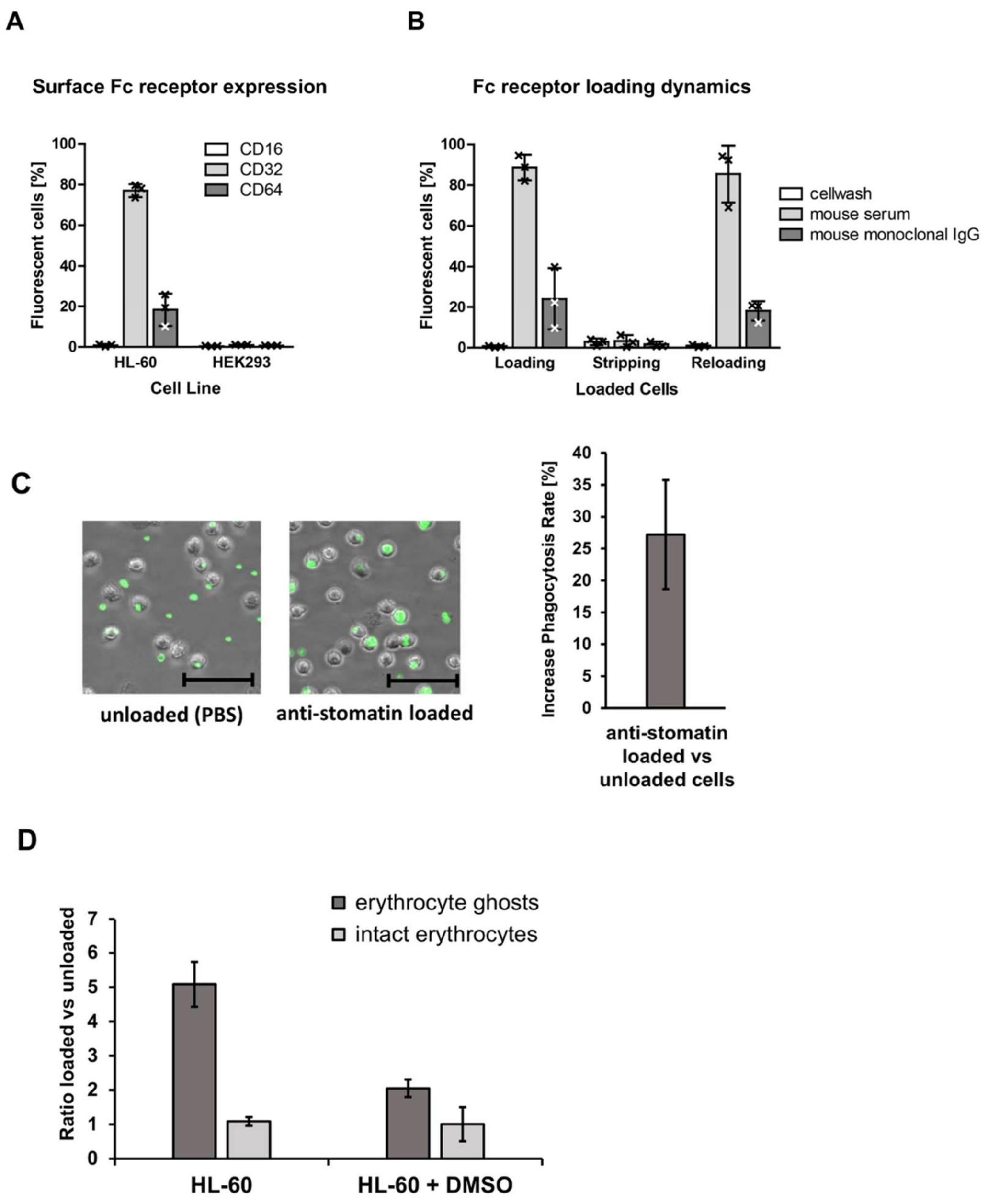

fig. S5. Expression and binding of Fcγ-receptors in cell lines and CD14+ monocytes. (**A**) Expression of Fcγ-receptors CD16, CD32 and CD64 in phagocyte cell lines HL-60 and in HEK293 cells. The results of three independent measurements are presented. (**B**) Robustness of binding of polyclonal mouse serum and murine monoclonal antibody to HL-60 cells during loading-stripping-reloading cycle. **(C)** CD14+ monocytes from five individuals were preloaded with a commercial anti-stomatin antibody or used un-loaded. Phagocytosis of stomatin-coated latex beads was measured. Representative microscopic images after 3 hours of phagocytosis at 37°C (left panel; scale bar= 50µm). The increase of phagocytosing cells of antibody loaded cells compared to unloaded cells are shown (right panel). (**D)** Ratio of the number phagocytosing cells in loaded (anti-stomatin) vs unloaded cells. Comparison of the phagocytosis of erythrocyte ghosts and intact erythrocytes by undifferentiated HL-60 cells and DMSO treated HL-60 cells

table S1. Abundance of monoclonal antibody-expressing B-VIREM cells in cytospin preparations s of PBMC from healthy individuals

Micrograph	Monocytes [n]	B-VIREM [n]	Abundance [%]
1	46	1	2.17
2	116	2	1.72
3	82	1	1.22
4	71	1	1.41
5	50	1	2.00
6	54	1	1.85
7	105	1	0.95
8	55	1	1.82
9	90	2	2.22
10	93	1	1.08
11	79	2	2.53
**Total**	**841**	**14**	
**Mean**			**1.73**
**SD**			**0.48**

table S2. Immunoglobulin heavy and light chain clonotypes (AA sequences and V(D)J-C gene segments) from HC, ERS and LRS samples

see attached “**Neumaier_TableS2.xls**”

table S3. Proteins identified in Vab1 immunoprecipitations. Top 30 proteins specifically precipitated by Vab1 in 24 immunoprecipitation reactions and identified by nano LC-MS/MS. Intracellular compartmental distribution (ICD) of proteins: M: membrane–associated proteins (grey); E: extracellular matrix; N: nuclear proteins; C: cytoplasmic proteins. All proteins reported to be integral part of erythrocyte membrane complexes are highlighted in bold.

Accession	Description	Count [n]	ICD
**P27105**	**Erythrocyte band 7 integral membrane protein (Stomatin)**	**15**	**M**
P47756	Isoform 2 of F-actin-capping protein subunit beta	9	S
P06899	Histone H2B type 1-J	8	N
Q16778	Histone H2B type 2-E	8	N
P02788	Isoform DeltaLf of Lactotransferrin	8	C
Q00610	Isoform 2 of Clathrin heavy chain 1	7	C
P12111	Isoform 2 of Collagen alpha-3(VI) chain	7	E
P50914	60S ribosomal protein L14	6	C
P12109	Collagen alpha-1(VI) chain	6	E
P52907	F-actin-capping protein subunit alpha-1	6	S
P12110	Isoform 2C2A' of Collagen alpha-2(VI) chain	6	E
Q01995	Transgelin	6	S
P15880	40S ribosomal protein S2	5	C
P36578	60S ribosomal protein L4	5	C
P04003	C4b-binding protein alpha chain	5	C
P23528	Cofilin-1	5	S
P21291	Cysteine and glycine-rich protein 1	5	C
P60981	Destrin	5	S
P60842	Eukaryotic initiation factor 4A-I	5	C
P09382	Galectin-1	5	C
Q8N257	Histone H2B type 3-B	5	N
Q96AC1	Isoform 2 of Fermitin family homolog 2	5	E
**P16157**	**Isoform Er8 of Ankyrin-1**	**5**	**M**
P17987	T-complex protein 1 subunit alpha	5	S
P07996	Thrombospondin-1	5	E
Q9ULV4	Coronin-1C	4	S
**P16452**	**Erythrocyte membrane protein band 4.2**	**4**	**M**
Q15746	Isoform 9 of Myosin light chain kinase_ smooth muscle	4	C
**P02549**	**Spectrin alpha chain erythrocytic 1**	**4**	**M**
**P11277**	**Spectrin beta chain erythrocytic**	**4**	**M**

table S4. Mass spectrometric protein identification in high molecular weight protein bands of immunoprecipitation reactions. Specific proteins, which were detected by mass spectrometry in high molecular weight bands of Vab1- or a commercial anti-stomatin antibody-immunoprecipitation reactions. All proteins reported to be integral part of erythrocyte membrane complexes are highlighted in bold.

Accession	Description	MW (kDa)	Vab1	Anti-stomatin
**P16157**	**Ankyrin-1**	**180**	**X**	**X**
**Q01484**	**Ankyrin-2**	**205**	**X**	
**Q12955**	**Ankyrin-3**	**203**	**X**	
Q13625	Apoptosis-stimulating of p53 protein 2	111		X
**P17858**	**ATP-dependent 6-phosphofructokinase liver type**	**85**	**X**	
**P02730**	**Band 3 anion transport protein**	**94**	**X**	
**P18577**	**Blood group Rh(CE) polypeptide**	**27**	**X**	**X**
P07384	Calpain-1 catalytic subunit	82	X	X
Q00610	Clathrin heavy chain 1	188	X	
**P27105**	**Erythrocyte band 7 integral membrane protein**	**32**	**X**	**X**
**P16452**	**Erythrocyte membrane protein band 4.2**	**69**	**X**	**X**
P48506	Glutamate--cysteine ligase catalytic subunit	73	X	
P62826	GTP-binding nuclear protein Ran	24	X	X
**P68871**	**Hemoglobin subunit beta**	**16**	**X**	
**P23276**	**Kell blood group glycoprotein**	**83**		**X**
**P11171**	**Protein 4.1**	**81**		**X**
**P11166**	**Solute carrier family 2 facilitated glucose transporter member 1**	**54**	**X**	**X**
**P02549**	**Spectrin alpha chain erythrocytic 1**	**280**	**X**	**X**
**P11277**	**Spectrin beta chain erythrocytic**	**246**	**X**	**X**
Q9Y4I1	Unconventional myosin-Va	212	X	
A4D1P6	WD repeat-containing protein 91	42		X

movie S1. Colocalization of simultaneously expressed CD32 and eGFP-Fc during intracellular vesicle transport

Live cell imaging video sequence of CD32-OFP (red) and eGFP-Fc (green) HEK293 double transfectants. Video sequence shows intracellular transport of both proteins to the cell surface with up to 20% of mixed fluorescence vesicles. Boxed area highlights the position of the close-up view in Movie S2.

movie S2. Close-up view of vesicle transport in CD32 and eGFP-Fc double tranfectants

Close-up view of video sequence of Movie S1.
